# Selective Production of Bio-Based Linear Alpha-Olefin from Wasted Fatty Alcohol on Al_2_O_3_ for Bio-Based Chemicals

**DOI:** 10.3390/polym13172850

**Published:** 2021-08-25

**Authors:** Hye-Jin Lee, Il-Ho Choi, Seung-Wook Kim, Kyung-Ran Hwang

**Affiliations:** 1 Energy Resource Upcycling Research Laboratory, Korea Institute of Energy Research, Daejeon 34129, Korea; qw9620qw@korea.ac.kr (H.-J.L.); 15choi@kier.re.kr (I.-H.C.); 2 Department of Chemical and Biological Engineering, Korea University, Seoul 136701, Korea; kimsw@korea.ac.kr

**Keywords:** bio-based alpha-olefins, long-chain alpha-olefins, waste fatty alcohol, dehydration, solvothermal method

## Abstract

The catalytic dehydration of a bio-based fatty alcohol was performed using Al_2_O_3_ prepared by solvothermal synthesis for selective production of long-chain linear-alpha-olefins (LAO). The effect of the synthesis temperature of alumina precursors on the dehydration of 1-octadecanol (C_18_H_38_O) was examined based on the textural properties and Lewis acid–base properties of the catalysts. Amorphous alumina synthesized at 325 °C showed the highest surface area (233.07 m^2^/g) and total pore volume (1.237 cm^3^/g) among the catalysts and the best dehydration results: 93% conversion, 62% selectivity of 1-octadecene (C_18_H_36_), and 89% LAO purity. This was attributed to the increased Al/O ratio and atomic concentration of surface O in alumina, which were important factors in the catalytic dehydration of 1-octadecanol through the synergistic catalysis of acid–base pairs. The produced bio-based LAO can be key intermediates for synthesis of oxo alcohols and poly-alpha-olefins, as alternatives to petroleum-based LAO to achieve carbon neutrality in chemical industry.

## 1. Introduction

Linear-alpha-olefins (LAO) are versatile building blocks to produce universal or high-value chemicals used in many industries such as co-monomer for polyethylene and polypropylene, plasticizer, cosmetics, pharmaceuticals and synthetic lubricants. The market size of LAO was estimated as USD 12.5 billion in 2016 and is expected to grow with the increasing demand (USD 19 billion in 2024) [1]. As the consumption of LAO increases, research on methods to produce LAO has also increased. There are several common processes to produce LAO including (I) catalytic oligomerization of fossil-based ethylene [2,3], and (II) dehydrogenation of synthetic n-alkanes from Fischer–Tropsch processing of syngas or direct conversion of syngas to olefins [4,5,6]. LAO can also be obtained through the thermal cracking of heavy hydrocarbons (III) such as wax or waste plastics based on petroleum [7,8,9]. However, in these processes, the chain length of olefins is not easily controlled, resulting in limited yields for medium (C_5_-C_12_) to long-chain (C_13+_) LAO [10,11]. In addition, because we are facing environmental problems such as global warming and the depletion of fossil resources, many researchers have recently focused on the production of bio-derived rather than petroleum-derived LAO.

**Figure 1:**
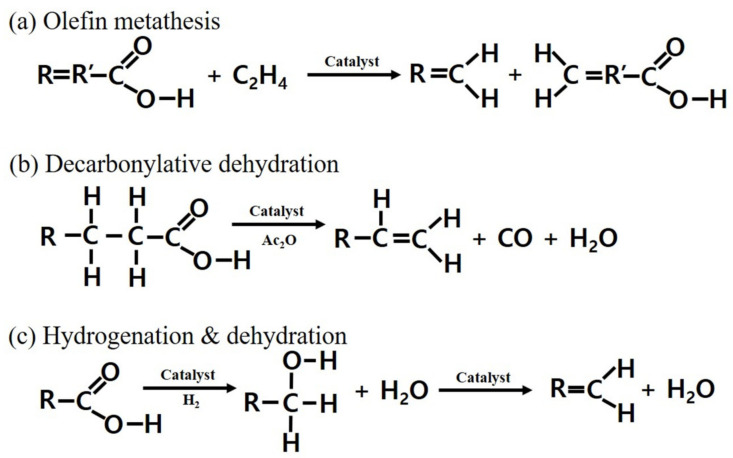
Various routes for bio-based LAO production; (**a**) olefin metathesis; (**b**) decarbonylative dehydration, and (**c**) hydrogenation and dehydration.

The bio-derived LAO can be obtained from wasted free fatty acids (FFAs), having a long-chain hydrocarbon structure and a carboxylic functional group. Currently, such low-cost feedstock containing significant amounts of FFAs is not suitable to produce bio-diesel due to the formation of soap and the increasing purification cost in the process of biodiesel production [12,13]. Thus, the wasted FFAs, which are generated from oil refining processes or the kraft paper-making industry in the form of palm sludge oil, palm fatty acid distillate, tall oil fatty acids, etc., appear to be suitable raw materials for the production of valuable chemicals [14,15,16]. Numerous routes to produce LAO based on biomass, as schematically illustrated in Figure 1, have been reported. Olefin metathesis (Figure 1a) is a highly selective route to produce LAO via self-metathesis of FFAs or cross-metathesis with FFAs and ethylene using ruthenium-based Grubbs’ catalysts [17]. However, only unsaturated FFAs are available for the metathesis and some limitations originating from homogenous catalysts still remain. Ru-complexes immobilized catalysts have thus been employed for olefin metathesis [18] and a two-step process including ethenolysis of intermediates obtained from decarboxylation of FFAs has also been suggested [19]. A decarbonylative dehydration reaction of FFAs (Figure 1b) has been explored to produce uncommon, odd-numbered LAO using Pd-based homogenous or heterogeneous catalysts. Some research groups achieved 66%–99% yield and 97% selectivity of LAO. However, a large amount of Pd catalyst (3 mol%) and an expensive solvent (1,3-Dimethyl-3,4,5,6-tetrahydro-2(1H)-pyrimidinone) are required to achieve the highest selectivity and best performance [20,21]. Although Liu et al. conducted decarbonylative dehydration of FFAs using a small amount of Pd catalyst (0.05 mol%) under a solvent-free condition and achieved satisfactory results (41%–80% yield, 80%–99% selectivity of LAO), stoichiometric amounts of acetic anhydride and selected phosphine ligands were still required [22]. Meanwhile, bio-based LAO can be synthesized by catalytic hydrogenation of FFAs into fatty alcohol, followed by dehydration of the fatty alcohol (Figure 1c). This route allows the synthesis of LAO without carbon loss regardless of the degree of saturation in fatty acids. Chen et al. conducted the hydrogenation of FFAs to corresponding fatty alcohol (99% yield) using a Ru-based catalyst followed by dehydration of the fatty alcohol to LAO (90% yield) using Al_2_O_3_ mixed with ThO_2_. The significant dehydration yield was due to the synergetic effect between (1) the favourable dehydration on the abundant Lewis acid sites (LAS) and Lewis base sites (LBS) on the nano-sized alumina and (2) the hindrance of further undesired isomerization of dehydrated LAO on thoria [23,24]. In fact, dehydration catalysts have been widely investigated for shorter alcohols such as ethylene production from dehydration of ethanol [25,26,27]. In particular, studies on dehydration of 1-alcohols using Al_2_O_3_ with rich LAS have been reported [28,29,30]. Kostestkyy et al. revealed that Al_2_O_3_ was the best catalyst for the dehydration of 1-propanol among metal oxides due to the stronger binding energy of alcohol on Al-LAS (binding energy = −133.0 kJ mol^−1^) than TiO_2_ and ZrO_2_ (−70.8 and −83.1 kJ mol^−1^, respectively) [31]. The dehydration of methanol and ethanol using Al_2_O_3_ with various crystalline phases showed that χ- and γ-mixed Al_2_O_3_ exhibited higher catalytic activity than pure χ- and γ- Al_2_O_3_ due to improved acidity [32,33]. In addition, alumina synthesized by the solvothermal method showed the best catalytic result in ethanol dehydration among various prepared alumina owing to the higher surface area originating from the small crystalline size and large amount of acid sites [34]. A γ-Al_2_O_3_ supported cobalt catalyst showed a more active and stable performance than the parent γ-Al_2_O_3_ in ethanol dehydration due to enhanced surface hydrophobicity and higher micro-pore acidity of Co/γ-Al_2_O_3_ [25]. Connor et al. investigated mixed C_2_-C_4_ alcohol dehydration over various acidic catalysts such as Zr-KIT-6, Al-MCM-41, HZSM-5, and SAPO-34 and suggested that Brønsted acid sites (BAS) were more active than LAS under the investigated conditions [26]. Unlike short alcohols, dehydration of C_4_ alcohol produced 1- and 2-butentes on the γ-Al_2_O_3_ or modified γ-Al_2_O_3_, meaning that a double-bond shift in olefins makes the selectivity of alpha-olefins low when using long-chain feedstock [27,35]. Moreover, diethyl ether was formed as a byproduct during ethanol dehydration on silica-alumina catalysts and the selectivities of ethylene and ether depended on the nature of Lewis sites with an alumina-like acid-basic neighbor or with a silica-like covalent neighbor in silica–alumina catalysts at relatively low reaction temperatures [36]. This means that the shift of the double bond at the alpha position of olefins (isomerization) and the side reaction (ether formation) should be considered when designing catalysts for selective dehydration of long-chain alcohols to LAO. However, there have been few reports on dehydration catalysts of long-chain alcohols derived from fatty acids for the selective production of long-chain LAO. Mingli et al. studied selective dehydration and hydro-processing for bio-jet fuel production from a medium-chain fatty alcohol and found that the acidic properties and structural features of the tested catalysts (ZSM-22, ZSM-5, and Al-MCF) were important to convert *n*-decanol into straight decene in the dehydration step. However, alpha-olefin (1-decene) was not observed in the straight decene groups that were mainly produced from the dehydration reaction using Al-MCF catalyst [37]. 

In this study, catalytic dehydration of a bio-derived long-chain fatty alcohol (1-octadecanol, C_18_H_38_O) was conducted using Al_2_O_3_ prepared by solvothermal synthesis for the selective production of bio-LAO (1-octadecene, C_18_H_36_). The effect of the synthesis temperature of Al_2_O_3_ catalyst on the catalytic dehydration of the long-chain fatty alcohol was discussed based on the textural properties and Lewis acid properties of the catalysts.

## 2. Experimental

### 2.1. Catalyst Preparation

Alumina catalysts were prepared by the solvothermal method reported by Janlamool et al. [33]. First, 6.25 g of aluminum isopropoxide (≥ 98%, Sigma-Aldrich, Saint Louis, MO, USA) and 25 mL of toluene (99.8%, Sigma-Aldrich, Saint Louis, MO, USA) were mixed in an autoclave (100 mL, Parr Instrument, Moline, IL, USA). After purging with N_2_ gas, the autoclave was heated to 275–350 °C (2.5 °C/min), maintained for two hours, and then cooled at room temperature. Four alumina precursors were synthesized at 275 °C, 300 °C, 325 °C, and 350 °C, respectively. The obtained product (alumina precursor) was washed several times with methanol (99%, Alfa Aesar, Ward Hill, MA, USA) and calcined at 600 °C for 6 h (10 °C/min). Alumina catalysts synthesized at different temperatures were denoted as according to the synthesis temperature in the autoclave. For example, alumina synthesized at 275 °C in the autoclave is denoted as A 275.

### 2.2. Catalyst Characterization 

The phase of the synthesized catalyst is important information for understanding the catalytic activity and was investigated in this study by X-ray diffraction (XRD) using a Rigaku Smartlab (Tokyo, Japan). The radiation source is Cu-α1 and the scan range is 10–80°. 

Surface properties of the synthesized catalysts including surface area, pore volume and pore distribution were confirmed by Brunauer–Emmett–Teller (BET) and Barrett–Joyner–Halenda (BJH) methods using a BELSORP-mini II (MictrotracBEL, Osaka, Japan).

The surface morphologies of the synthesized catalysts and the local structures of catalysts were examined by using a high-resolution scanning electron microscope (SEM, SU8230, Hitachi, Tokyo, Japan) and a field emission-transmission electron microscope (FE-TEM, JEM-2100F, JEOL, Tokyo, Japan), respectively.

An X-ray photoelectron spectrometer (XPS) is widely used as powerful analysis equipment for investigating elemental composition and oxidation state. In order to analyze the surface characteristics of the synthesized catalysts, an XPS analysis was conducted using a Thermo Scientific K-alpha+ (Waltham, MA, USA) equipped with a radiation source of a micro-focused monochromatic Al Kα X-ray beam (binding energy = ~1350 eV). The charging shifts of the spectra were calibrated using the C_1s_ peak at 284.45 eV.

In order to confirm the type and strength of the acid site, diffuse reflectance infrared Fourier transform spectroscopy (DRIFTS, Thermo Scientific Nicolet iS50+, Waltham, MA, USA) with the adsorption–desorption technique of pyridine (Sigma-Aldrich, Saint Louis, MO, USA) as a probe molecule was conducted. The sample was pretreated by heating to 380 °C for 1 h with He 60 mL/min. Pyridine gas adsorption was then conducted using 60 mL/min of 1 vol% pyridine/He at 100 °C for 1 h. After pyridine adsorption, the sample was purged by 60 mL/min of He gas to remove physically adsorbed pyridine. After purging at 50 °C, the signal was obtained by desorbing pyridine at 350 °C.

Thermogravimetric analysis (TGA) of the spent catalyst was carried out using a TGA Q500 (TA instruments, New Castle, DE, USA) with air. Prior to analysis, the used catalyst was washed with acetone (Alfa Aesar, Ward Hill, MA, USA) followed by drying at 70 °C overnight. 

### 2.3. Dehydration of Long-Chain Fatty Alcohol and Product Analysis

Dehydration of 1-octadecanol was carried out with the prepared catalysts. Representative reaction conditions are as follows. 0.1 g of catalyst, 1 g of 1-octadecanol (99%, Sigma-Aldrich, Saint Louis, MO, USA) and 20 mL of dodecane (≥99.0%, Sigma-Aldrich, Saint Louis, MO, USA) were loaded into a batch reactor (100 mL, Parr Instrument, Moline, IL, USA). After purging with N_2_ gas, the reactor was heated to 300 °C at N_2_ 15 bar pressure. It was then stirred at 500 rpm and maintained at 300 °C for 2 h. After cooling to room temperature, 0.1 mL of n-decane (>99.0%, Tokyo Chemical Industry, Tokyo, Japan) as an internal standard material for quantitative analysis was added in the obtained product. The product was then analyzed using a gas chromatograph (GC, Shimadzu GC2010 plus, Kyoto, Japan) equipped with a Restek Rtx-5 capillary (60 m × 0.32 mm × 0.25 μm, Centre County, PA, USA) and a flame ionization detector. In addition, experiments with different conditions were conducted to examine the influence of the reaction parameters (temperature, time, and ratio of reactant and catalyst) on the dehydration of 1-octadecanol.

In order to determine the catalyst activity, parameters such as the conversion of 1-octadecanol, selectivity of 1-octadecene, yield of 1-octadecene, and purity of 1-octadecene were calculated by using Equations (1)–(4), respectively.

(1) $$\mathrm{Conversion}\left(\%\right)=\frac{\mathrm{amount}\;\mathrm{of}\;\mathrm{reacted}\;1-\mathrm{octadecanol}}{\mathrm{amount}\;\mathrm{of}\;\mathrm{initially}\;\mathrm{added}\;1-\mathrm{octadecanol}}\times 100$$

(2) $$\mathrm{Selectivity}\left(\%\right)=\frac{\mathrm{amount}\;\mathrm{of}\;\mathrm{produced}\;1-\mathrm{octadecene}}{\mathrm{amount}\;\mathrm{of}\;\mathrm{reacted}\;1-\mathrm{octadecanol}}\times 100$$

(3) $$\mathrm{Yield}\;\left(\%\right)=\frac{\mathrm{amount}\;\mathrm{of}\;\mathrm{produced}\;1-\mathrm{octadecene}}{\mathrm{amount}\;\mathrm{of}\;\mathrm{initially}\;\mathrm{added}\;1-\mathrm{octadecanol}}\times 100$$

(4) $$\mathrm{Purity}\left(\%\right)=\frac{\mathrm{amount}\;\mathrm{of}\;\mathrm{produced}\;1-\mathrm{octadecene}}{\mathrm{amount}\;\mathrm{of}\;\mathrm{produced}\;\mathrm{octadecenes}}\times 100$$

The produced octadecenes in the term “purity (%)” include 1-octadecene and its isomers.

Di-stearyl ether can be generated during the dehydration reaction of 1-octadecanol as a byproduct. Simulated distillation gas chromatography (SIMDIS) is adopted as an indirect method to observe di-stearyl ether, which has a high boiling point. The analysis was conducted using the ASTM D 2887 standard method.

## 3. Results and Discussion

### 3.1. Characteristics of Catalysts

**Figure 2:**
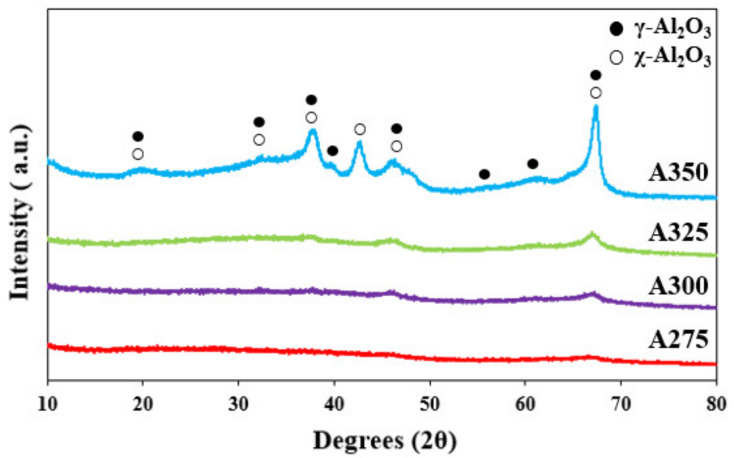
XRD patterns of the alumina samples A275, A300, A325, and A350 prepared at 275 °C, 300 °C, 325 °C, and 350 °C, respectively.

XRD profiles of the catalysts prepared by the solvothermal synthesis are depicted in Figure 2. No peaks were observed in the XRD pattern of A275 catalyst and some significant peaks started to appear from A325. However, A300 and A325 catalysts are essentially amorphous even though weak peaks were observed in the XRD pattern [38]. 19.4°, 32.5°, 37.48°, 39.3°, 45.8°, 60.7°, and 66.9° corresponded to the diffraction planes of γ-Al_2_O_3_ (JCPDS 00-050-0741) [39], and 18.5°, 31.0°, 37.3°, 42.6°, 46.3°, and 67.0° matched the diffraction planes of χ-Al_2_O_3_ (JCPDS 00-013-0373) [40]. Finally, the clearer γ-χ mixed phase of alumina appeared in the XRD pattern of the A350 catalyst. This meant that amorphous oxide or crystalline oxide could be synthesized according to the temperatures for solvothermal synthesis, even though all alumina precursors were calcined at 600 °C for 6 h.

The BET surface area and pore properties of the prepared catalysts are summarized in Table 1. It can be seen that the surface area of the prepared alumina catalysts, except A325, decreased with increasing synthesis temperature of the alumina precursors. A325 showed the highest surface area (233.07 m^2^/g) and total pore volume (1.237 cm^3^/g) among the catalysts. In the preparation of the alumina precursors by the solvothermal method, toluene solvent exists as a supercritical fluid in the autoclave above 325 °C. The increased homogeneity and reactivity between aluminum isopropoxide and toluene in the supercritical conditions appeared to lead to the increase in surface area and pore volume of A325 catalyst. However, the surface area of A350 was smaller than that of A325 and the worst among the prepared catalysts. This appears to be attributable to the formation of a crystalline phase, as shown in the XRD pattern (Figure 2). 

**Figure 3:**
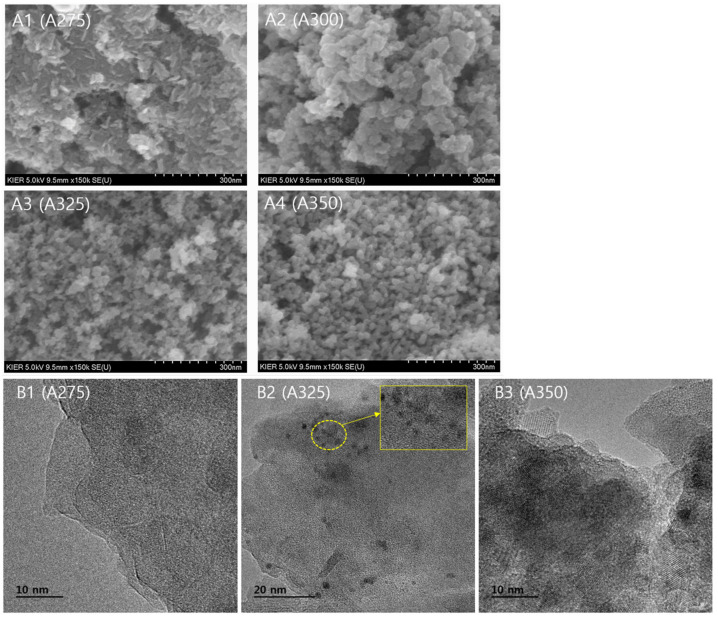
SEM (**A1**–**A4**) and FE-TEM (**B1**–**B3**) images of the alumina samples A275, A300, A325, and A350.

The surface morphologies and the local structures of the prepared alumina catalysts are shown in Figure 3. As can be seen in SEM images (A1–A4), the surface morphologies of the prepared alumina catalysts were significantly changed depending on the synthesis conditions; the nanoparticles were interconnected with each other and the spherical nanoparticles with uniform appearance appeared with the increase in synthesis temperature. Amorphous oxide was observed in FE-TEM images of A275 (B1) and A325 (B2), but there were some nanocrystallites within the amorphous matrix (B2). Lattice fringes of nanocrystallites were clearly visible in a FE-TEM image of A350 (B3), which is consistent with the results of XRD. 

**Figure 4:**
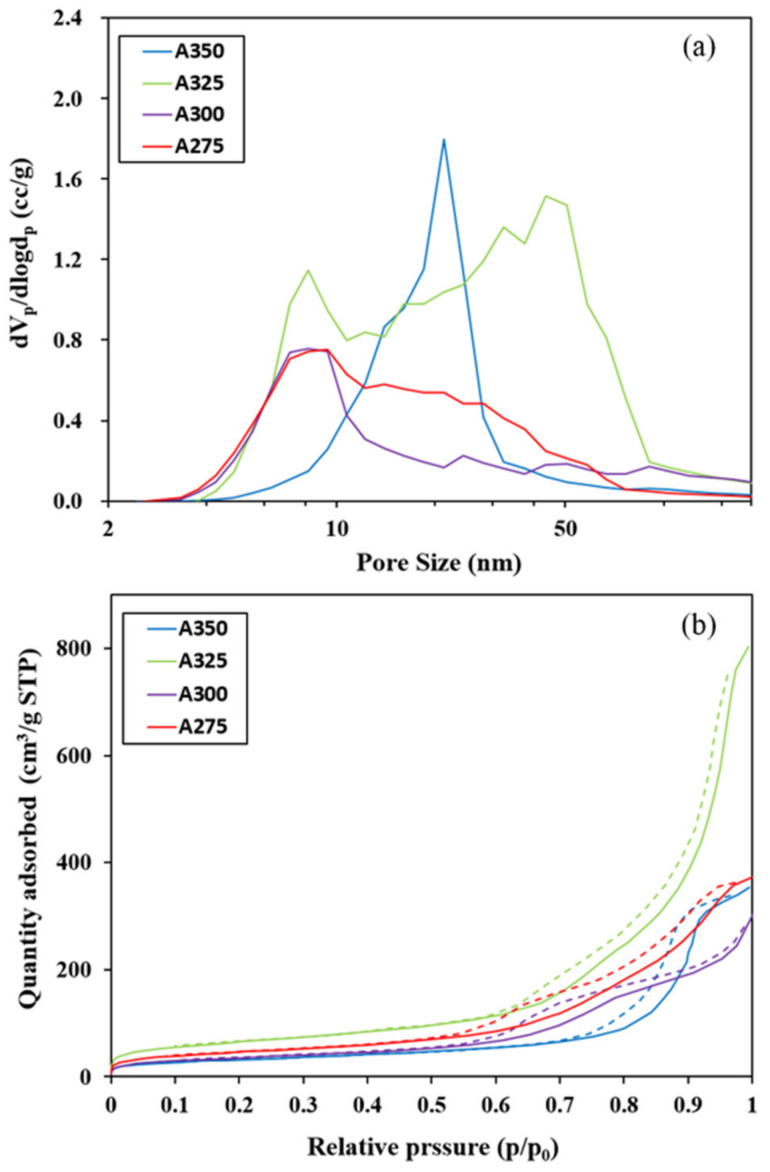
(**a**) Pore size distribution profiles; (**b**) N_2_ adsorption-desorption isotherms of the alumina catalysts A275, A300, A325, and A350.

Figure 4 shows the pore size distribution profiles (a) and N_2_ adsorption–desorption isotherms (b) of the alumina catalysts A275, A300, A325, and A350 synthesized at 275, 300, 325, and 350 °C, respectively. In the case of the pore size distribution of the catalysts (a), A325, having a large total pore volume (Table 1), has not only meso-pores but also macro-pores over 50 nm. A350 has a relatively specific peak of about 21 nm, indicating that pores are made uniformly, unlike others. Based on the results of N_2_ adsorption–desorption isotherms (b), only A350 had a H1-type hysteresis loop according to the classification of IUPAC. This indicates that A350 has a narrow pore size distribution because most of the pores are surrounded by uniform spherical particles. It is difficult to classify all other catalysts, except A350, according to the IUPAC classification but A325 catalyst had around a twofold higher adsorbed amount than the other catalysts. 

**Figure 5:**
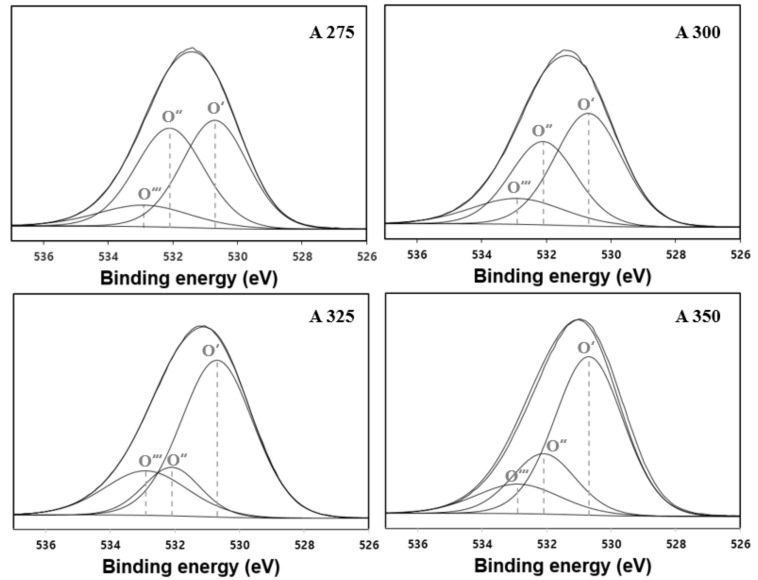
The XPS deconvolution results of O_1s_ of the alumina catalysts A275, A300, A325, and A350.

It is reported that the dehydration reaction of fatty alcohol progresses through the synergistic catalysis of acid–base pairs on the catalyst surface, where the yield of LAO is dependent on the acid–base ratio of alumina [41]. Thus, an XPS analysis was carried out to obtain information on the oxidation states and element contents of the alumina catalysts. XPS spectra of O_1s_ and data of Al_2p_ and O_1s_ are shown in Figure 5 and Table 2, respectively. The peak at 530.7 eV (labeled O′_1s_) corresponds to oxygen in the alumina and the peaks at 532.1 eV (labeled O″_1s_) and at 532.9 eV (labeled O‴_1s_) might correspond to the OH groups and water on the surface of alumina, respectively [33]. By fitting the O_1s_ peak with three sets of oxygen species, we found that the surface O (O′ _1s_) increased with increasing synthesis temperature of the catalysts, whereas OH (O″_1s_) and H_2_O (O‴_1s_) decreased (summarized in Table 2). Moreover, as the synthesis temperature increased, the Al to O ratio also increased from 0.56 to 0.68, reaching the composition of the Al_2_O_3_ crystalline state (0.67). Kostestkyy et al. reported the importance of surface O and/or OH groups of the oxides as active centers in Lewis acid-catalyzed dehydration reaction of alcohols [31]. Based on their calculation of the energy barrier for alcohol dehydration via a concerted E2 elimination mechanism, when a hydroxyl group in the alcohol adsorbs on the aluminum (Lewis acid center) of alumina catalyst, β-hydrogen more strongly adsorbs on surface O than the OH group owing to the lower dehydration barrier, which originates from the more basic property of surface O. Therefore, it can be expected that the increased Al/O ratio and atomic concentration of surface O in the prepared catalysts promote the catalytic behavior of dehydration of fatty alcohol through the synergistic catalysis of acid–base pairs.

**Figure 6:**
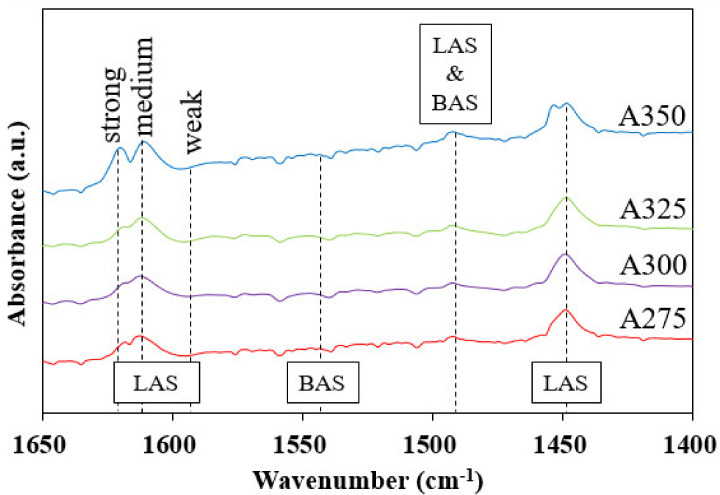
Pyridine-FTIR spectra of the synthesized alumina catalysts A275, A300, A325, and A350.

As mentioned above, the dehydration of alcohol is initiated by attracting –OH group of alcohol to the acid site. Figure 6 demonstrates pyridine-FTIR spectra of the synthesized alumina catalysts A275, A300, A325, and A350 in the range 1400–1650 cm^−1^ to obtain information on acid centers. The bonding between pyridine and LAS or BAS can be identified at 1450 or 1545 cm^−1^, and the band at around 1490 cm^−1^ indicates LAS and BAS simultaneously [29,42,43]. In Figure 6, all alumina catalysts showed peaks at 1450 and around 1490 cm^−1^, which indicates that only LAS exists in the prepared catalysts. Additionally, the bands at 1622, 1612, and 1594 cm^−1^, assigned to coordinately unsaturated Al^3+^ cations in alumina, are recognized as a strong, medium, and weak LAS [43,44,45]. The dehydration of alcohol over alumina catalysts thus clearly progresses on LAS (metal center), not BAS. 

### 3.2. Dehydration Reaction of 1-Octadecanol

**Figure 7:**
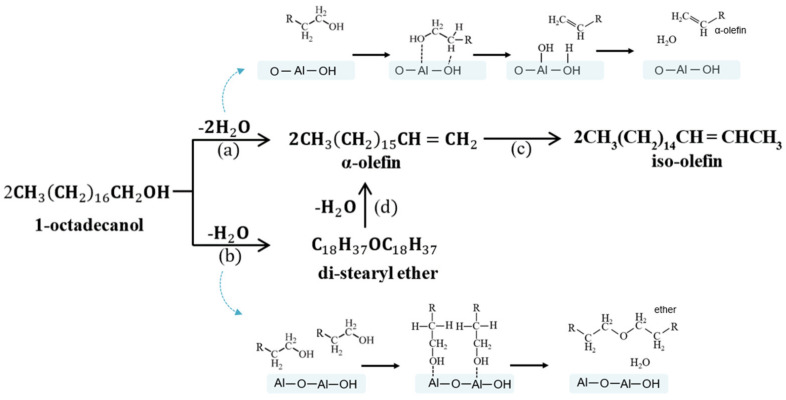
Possible dehydration reaction routes of 1-octadecanol: (**a**) intramolecular route; (**b**) intermolecular way; (**c**) re-adsorption of LAO on LAS on the surface of alumina, and (**d**) di-stearyl ether intermediate decomposition into LAO and water.

Figure 7 delineates the possible dehydration reaction routes of 1-octadecanol. The pathway (a, intramolecular route) is conducted via direct dehydration of one alcohol molecule adsorbed on alumina surface. Conversely, the pathway (b, intermolecular way) occurs via two alcohol molecules that co-adsorbed in nearby sites on the alumina surface [33]. The produced di-stearyl ether intermediate can be decomposed into α-olefin and water (d). Here, the α-olefin formation pathways require an acid–base pair consisting of Al and surface O (or OH) [41]. The isomerization of α-olefin to iso-olefin (linear internal olefin, LIO) (c) can take place through re-adsorption of α-olefin on LAS on the surface of alumina [28].

**Figure 8:**
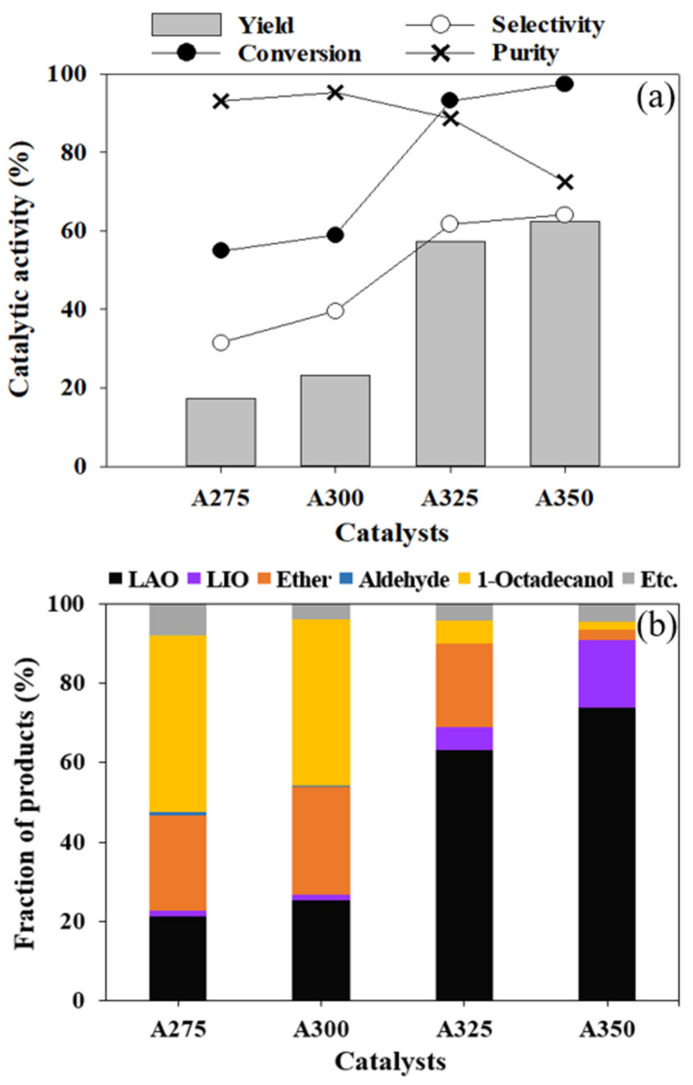
The results of 1-octadecanol dehydration (**a**) and product distribution after dehydration reaction of 1-octadecanol; (**b**) reaction condition: 2 h, 300 °C, 500 rpm, N_2_ 15 bar, 1 g of 1-octadecanol, 0.1 g of catalyst, 20 mL of n-dodecane.

**Figure 9:**
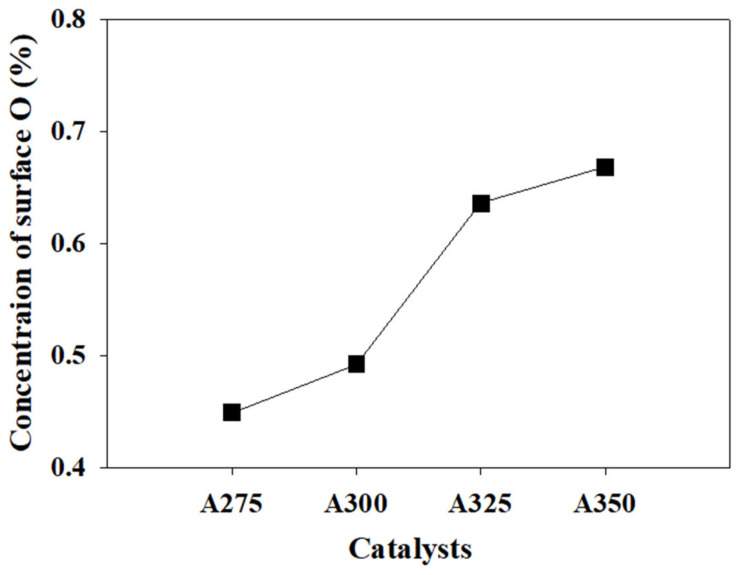
Concentration of surface O as a function of catalyst synthesis temperature, drawn from the XPS data (Table 2).

Figure 8 shows the results of the dehydration reaction of 1-octadecanol on the prepared catalysts. As shown in Figure 8a, the conversion of 1-octadecanol and selectivity and yield of 1-octadecene increased with an increase in catalyst synthesis temperature. This trend is accordant with the concentration of surface O in the catalysts, described in Figure 9. That is, the substantially enhanced conversion from using the A325 catalyst resulted from the increased Al/O ratio and the remarkably increased surface O in the acid–base pair catalytic system. However, the purity decreased with the catalyst synthesis temperature, indicating that isomerization of LAO to LIO occurred through re-adsorption of α-olefin on LAS on the surface. Remarkably strong bands at 1622–1594 cm^−1^ (LAS) of the A350 catalyst, as shown in Figure 6 (pyridine-FTIR), reflect that A350 has catalytic activity for isomerization reaction of the produced LAO as well as dehydration of alcohol. The product distribution after the dehydration reaction of 1-octadecanol is described in Figure 8b. Di-stearyl ether (C_36_H_74_O) cannot be identified by GC due to the high boiling point. Thus, SIMDIS was adopted as an indirect method to observe the product distribution including di-stearyl ether. A large amount of di-stearyl ether was produced when using A275, A300, and A325 catalysts and the content of di-stearyl ether apparently decreased, especially when using the A350 catalyst. This indicates that the produced ether was decomposed into LAO and water in the acid–base pair catalytic system, as mentioned above.

**Figure 10:**
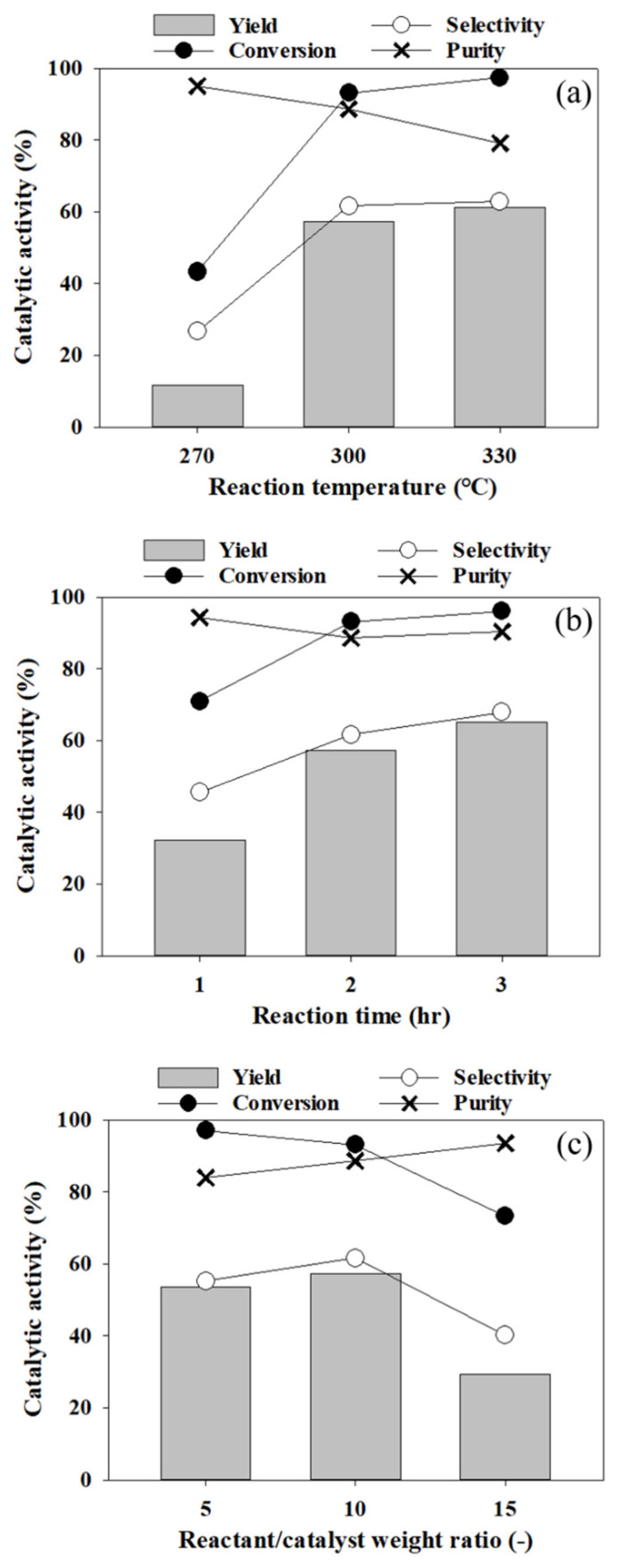
The results of 1-octadecanol dehydration according to various conditions: (**a**) 2 h, 500 rpm, N_2_ 15 bar, 1 g of 1-octadecanol, 0.1 g of catalyst (A325), 20 mL of n-dodecane; (**b**) 300 °C, 500 rpm, N_2_ 15 bar, 1 g of 1-octadecanol, 0.1 g of catalyst (A325), 20 mL of n-dodecane, and (**c**) 2 h, 300 °C, 500 rpm, N_2_ 15 bar, 1 g of 1-octadecanol, 0.067–0.200 g of catalyst (A325), 20 mL of n-dodecane.

Considering conversion, yield, and purity, the A325 catalyst was selected to examine the influence of reaction parameters (temperature, time, and reactant/catalyst ratio) on the catalytic dehydration reaction of 1-octadecanol and the results are shown in Figure 10. Reaction temperature and reaction time positively affected 1-octadecanol conversion and LAO yield. In terms of LAO purity, it was more sensitive to reaction temperature rather than reaction time. Multiple linear regression was conducted to evaluate the effect of independent variables (temperature, time, and reactant/catalyst ratio) on LAO purity. Two variables (temperature and reactant/catalyst ratio) statistically significantly predicted LAO purity (*p* < 0.014, adj. *R^2^* = 0.776). Coefficients summarized in Table 3 demonstrate that as the reaction temperature increases by 1 °C, the purity decreases by 0.27%, whereas there is an increase in the purity of 0.95% as the reactant/catalyst ratio increases by 1. A standardized coefficients, *β*, indicate that the effect of the reaction temperature on the purity is relatively greater than that of the reactant/catalyst ratio.

The spent alumina catalyst (A350) was washed with acetone, dried at 70 °C overnight, and used for the second dehydration testing of 1-octadecanol. Conversion and LAO yield decreased by 9.1% and 14%, respectively, compared to the initial conversion. The TGA result showed that the used catalyst had two significant weight loss peaks: 5.2 wt% at 142.5 °C and 11.9 wt% at 325.5 °C with a shoulder at 490 °C. The spent catalyst was therefore regenerated by recalcining the catalyst under air at 600 °C for 6 h and consequently, the regenerated catalyst restored its initial catalytic activity.

## 4. Conclusions

The catalytic dehydration of a bio-based fatty alcohol (1-octadecanol, C_18_H_38_O) was performed using Al_2_O_3_ prepared by solvothermal synthesis for selective production of the long-chain LAO. Alumina crystallization was determined by the synthesis temperature of alumina precursors and the clearer γ-χ mixed phase of alumina appeared in the XRD pattern of the A350 catalyst. A325 showed the highest surface area (233.07 m^2^/g) and total pore volume (1.237 cm^3^/g) among the catalysts, owing to the increased homogeneity and reactivity between aluminum isopropoxide and toluene. The Al/O ratio and atomic concentration of surface O in the prepared alumina increased with the synthesis temperature of alumina precursors, especially from the A325 catalyst, which served as important factors in the catalytic dehydration of 1-octadecanol through the synergistic catalysis of acid–base pairs. It was found that 93% conversion, 62% selectivity of 1-octadecene (C_18_H_36_), and 89% LAO purity were obtained from the dehydration of 1-octadecanol with the A325 catalyst. The A350 catalyst, having remarkably strong Lewis acid sites, decomposed the produced di-stearyl ether intermediate (C_36_H_74_O) into LAO but showed the lowest LAO purity, owing to the activity for isomerization of LAO to linear internal olefins on Lewis acid sites. Reaction temperature and reaction time positively affected 1-octadecanol conversion and LAO yield. However, the purity of LAO was more sensitive to the reaction temperature than the reactant/catalyst ratio. 

The bio-derived long-chain LAO as alternatives are key intermediates, that can be applied immediately to the synthesis of oxo alcohols and the poly-alpha-olefins. As the global market value of LAO is expected to grow due to increasing demand of plastics, detergents, and synthetic lubricants, replacing petro-based LAO with bio-based LAO is, therefore, very important in terms of environmental protection, carbon neutrality, and sustainability.

## Figures and Tables

**Table 1:** Textural properties (surface area, pore volume, and pore distribution) of the alumina catalysts A275, A300, A325, and A350.

Catalysts	BET Surface Area (m^2^/g)	Pore Volume (cm^3^/g)	Average Pore Diameter (nm)
A275	165.12	0.574	7.2
A300	126.56	0.432	7.2
A325	233.07	1.237	8.2
A350	114.02	0.541	21.3

**Table 2:** XPS data of Al_2p_ and O_1s_ of the alumina catalysts A275, A300, A325, and A350.

Catalysts	Binding Energy for Al_2p_ (eV)	Binding Energy for O_1s_ (eV)	Atomic Concentration (%)		Atomic Ratio (-)	O 1s Peak Area Fraction (%O_1s_)			O 1s Atomic Concentration (%)		
			Al 2p	O 1s	Al/O	H_2_O	OH	O	H_2_O	OH	O
A275	74.38	531.74	35.76	64.24	0.56	0.123	0.428	0.449	7.9	27.47	28.87
A300	74.38	531.69	37.49	62.51	0.6	0.154	0.354	0.492	9.61	22.12	30.77
A325	74.25	531.49	37.39	62.61	0.6	0.21	0.154	0.636	13.12	9.66	39.82
A350	74.36	531.42	40.42	59.58	0.68	0.13	0.202	0.668	7.76	12.01	39.8

**Table 3:** Coefficients estimated from multiple regression analysis.

	Unstandardized Coefficients		Standardized Coefficients	T(*p*)	Toler-ance(*TOL*)	Variance Inflation Factor (*VIF*)
	B	Std. Error	*Β*			
(constant)	163.26	17.81	0.00	9.17	1.0	1.0
Temperature	−0.27	0.06	−0.78	−4.65 **	1.0	1.0
Reactant/catal. ratio	0.95	0.34	0.47	2.78 *	1.0	1.0
*F(p)*	10.26^*^					
Adjusted *R^2^*	0.776					

* significant (*p*) < 0.05 and ** *p* < 0.01.

## Data Availability

The data presented in this study are available on request from the corresponding author.
